# First in vitro measurement of VHEE relative biological effectiveness (RBE) in lung and prostate cancer cells using the ARES linac at DESY

**DOI:** 10.1038/s41598-024-60585-7

**Published:** 2024-05-13

**Authors:** Hannah C. Wanstall, Florian Burkart, Hannes Dinter, Max Kellermeier, Willi Kuropka, Frank Mayet, Thomas Vinatier, Elham Santina, Amy L. Chadwick, Michael J. Merchant, Nicholas T. Henthorn, Michael Köpke, Blae Stacey, Sonja Jaster-Merz, Roger M. Jones

**Affiliations:** 1 Department of Physics and Astronomy, Faculty of Science and Engineering, The University of Manchester, Oxford Road, Manchester, M13 9PL UK; 2 Manchester Academic Health Science Centre, The Christie NHS Foundation Trust, Wilmslow Road, Manchester, M20 4BX UK; 3 Daresbury Laboratory, The Cockcroft Institute, Daresbury, Warrington, WA4 4AD UK; 4 Deutsches Elektronen Synchrotron (DESY), Notkestrasse 85, 22607 Hamburg, Germany; 5 Division of Cancer Sciences, Faculty of Biology, Medicine and Health, School of Medical Sciences, The University of Manchester, Oxford Road, Manchester, M13 9PL UK

**Keywords:** Biological physics, Cancer, Cell biology, Medical research, Oncology

## Abstract

Very high energy electrons (VHEE) are a potential candidate for radiotherapy applications. This includes tumours in inhomogeneous regions such as lung and prostate cancers, due to the insensitivity of VHEE to inhomogeneities. This study explores how electrons in the VHEE range can be used to perform successful in vitro radiobiological studies. The ARES (accelerator research experiment at SINBAD) facility at DESY, Hamburg, Germany was used to deliver 154 MeV electrons to both prostate (PC3) and lung (A549) cancer cells in suspension. Dose was delivered to samples with repeatability and uniformity, quantified with Gafchromic film. Cell survival in response to VHEE was measured using the clonogenic assay to determine the biological effectiveness of VHEE in cancer cells for the first time using this method. Equivalent experiments were performed using 300 kVp X-rays, to enable VHEE irradiated cells to be compared with conventional photons. VHEE irradiated cancer cell survival was fitted to the linear quadratic (LQ) model (R^2^ = 0.96–0.97). The damage from VHEE and X-ray irradiated cells at doses between 1.41 and 6.33 Gy are comparable, suggesting similar relative biological effectiveness (RBE) between the two modalities. This suggests VHEE is as damaging as photon radiotherapy and therefore could be used to successfully damage cancer cells during radiotherapy. The RBE of VHEE was quantified as the relative doses required for 50% (D_0.5_) and 10% (D_0.1_) cell survival. Using these values, VHEE RBE was measured as 0.93 (D_0.5_) and 0.99 (D_0.1_) for A549 and 0.74 (D_0.5_) and 0.93 (D_0.1_) for PC3 cell lines respectively. For the first time, this study has shown that 154 MeV electrons can be used to effectively kill lung and prostate cancer cells, suggesting that VHEE would be a viable radiotherapy modality. Several studies have shown that VHEE has characteristics that would offer significant improvements over conventional photon radiotherapy for example, electrons are relatively easy to steer and can be used to deliver dose rapidly and with high efficiency. Studies have shown improved dose distribution with VHEE in treatment plans, in comparison to VMAT, indicating that VHEE can offer improved and safer treatment plans with reduced side effects. The biological response of cancer cells to VHEE has not been sufficiently studied as of yet, however this initial study provides some initial insights into cell damage. VHEE offers significant benefits over photon radiotherapy and therefore more studies are required to fully understand the biological effectiveness of VHEE.

## Introduction

Very high energy electron (VHEE) radiotherapy is typically described as electrons accelerated to energies in the 100–250 MeV range. The idea of using VHEE as a novel radiation to treat cancer was first developed by Desrosiers et al.^1^ over 20 years ago. Since this initial investigation, interest in VHEE as a novel radiotherapy technique has expanded, with the development of the first VHEE radiotherapy device announced in 2022, as a collaboration between CERN, Centre Hospitalier Universitaire Vaudois (CHUV) and industry partner THERYQ. The radiotherapy device is expected to be operational by 2024, with first clinical trials planned for 2025^2,3^. The VHEE radiotherapy device under development will deliver VHEE at ultra-high dose rates (UHDR) with the aim to deliver FLASH radiotherapy, a novel treatment that uses UHDR to spare healthy tissue. A key driver for this collaboration is that VHEE is thought to be an ideal candidate for FLASH radiotherapy due to the fast and efficient dose delivery capabilities of electrons.

Another benefit of VHEE would be potential advantages during irradiation of cancers located in inhomogeneous regions, such as lung and prostate^4,5^. This is due to VHEE having relative insensitivity to regions of varying densities, such as air pockets, in comparison to the dose deposited as a result of irradiation with photons or protons. Increasing electron energy results in a reduced penumbra^1,4^ and therefore reduced dose scatter through a patient, indicating higher beam energies could be ideal for radiotherapy. Comparisons between VMAT and VHEE treatment plans indicate that VHEE resulted in similar or superior dose distribution for cases that include lung and prostate cancers^6^.

Electron energy in the range of 100–250 MeV significantly increases penetration depth so that the treatment of deep seated tumours would be possible^4^. Electrons in the (6–20 MeV) energy range have a long history of being used in the clinic for various superficial radiotherapy treatments due to their lower energy and therefore there reduced penetration^7,8^.

Although significant progress has been made in the development of VHEE for radiotherapy treatment, one aspect with extremely limited data is radiobiology. To our knowledge, there is no published in vitro or in vivo data at the time of writing. First investigations into the biological effectiveness of VHEE have been completed using theoretical models and by measuring damage to plasmid DNA, as a simplistic biological model. These studies aim to quantify the relative biological effectiveness (RBE) of VHEE. RBE is defined as the ratio of two doses where the radiation of interest (VHEE) is compared to a reference modality^9^, typically 250 kVp X-rays. The first experimental investigation into VHEE measures single strand (SSB) and double strand (DSB) DNA breaks to pBR322 plasmid DNA in response to 100–200 MeV electron irradiation (in comparison to ^60^Co X-rays)^10^. RBE of VHEE was measured to be ~ 1.1–1.2, where yield of DSBs was the biological measure. This result was validated in response to 35 MeV electrons in an identical plasmid model, with SSB yield as the biological endpoint^11^. Monte Carlo simulations of VHEE have predicted their RBE to be ~ 1.0, with no significant difference relative to photons^12^.

If VHEE radiotherapy is to be implemented clinically, characterisation of VHEE RBE is critical in both cancer and healthy tissue. As the field progresses, it is expected that RBE measurements will be completed across both in vitro and in vivo models, to fully understand the interaction of this novel radiotherapy modality with biological matter, ranging from DNA to tissues. An important step in this process is an RBE measurement of cells in vitro. This will provide initial measurements that can be used to direct in vivo studies, as well as patient research and treatment.

Currently, investigative studies into VHEE radiobiology are extremely limited. One of the most critical obstacles is the lack of biological facilities in close proximity to VHEE accelerators. The overlap of physics and biology research means that very few facilities have the required infrastructure for good aseptic technique to support repeatable radiobiology. The experiment was therefore chosen to be completed at ARES, DESY due to the availability of facilities in close proximity to the VHEE beamline.

To produce radiobiology results with statistical significance, a minimum of three repeats of any in vitro experiment is typically required, with all samples undergoing identical experimental conditions. The repetition of sample irradiation can require considerable durations of VHEE beam time, which is typically competitive and limited. The ability to replicate exact irradiation conditions can present a problem for VHEE accelerators. The machine needs to be highly stable between irradiations and ideally maintain consistent beam energy, shape and alignment throughout all experimental repeats. This can provide a problem in facilities with a rotation of users, as beam conditions will typically be altered frequently and replicating a very specific previous set of conditions can take significant additional time. This is a symptom of current VHEE research machines. Development into clinical use necessitates beam consistency, which will improve beam stability and functionality, improving the feasibility of radiobiology experiments.

Another hurdle with current VHEE accelerators in a research setting is achieving the required field size, which in most cases will be significantly larger than the electron beam. Electron beam size varies between accelerators. At DESY’s ARES facility we used a Gaussian beam with σ ≃ 1.3 mm. Current VHEE accelerators including the CERN linear accelerator for research (CLEAR), the sources for plasma accelerators and radiation compton with lasers and beams (SPARC) and the next linear collider test accelerator (NLCTA) have a Gaussian beam within a range of σ ≃ 1–5 mm^13^. This is considerably smaller than the irradiation area required for most typical in vitro experiments where irradiations are commonly performed in cell culture flasks with cells adherent to the flask surface (ranging between 25 and 225 cm^2^ culture area) or well plates (typical area of ~ 13.0 × 8.5 cm). Approaches to increase beam size include using materials such as foils or water to scatter the beam, otherwise pencil beam scanning can provide an overlapping dose profile. Both methods increase irradiation time of the sample which can be problematic when short time points post-irradiation are being investigated. This is particularly an issue when trying to obtain ultra-high dose rates. One way to achieve these dose rates would be by using traditional scattering methods. If spot-scanning methods were to be used to achieve ultra-high dose rates, an extremely high scanning speed (estimated at ~ 5.1 m/s) would be required to create dose rates within the FLASH range^13^.

The consideration of all these features can make a successful radiobiology experiment with VHEE more difficult, expensive and lengthy than experiments with more established modalities such as photons or protons. Fortunately, the current interest into using VHEE for medical applications has yielded several VHEE accelerator development projects worldwide, giving an optimistic outlook on the suitability of VHEE accelerators for radiobiology research. To progress the translational pathway for VHEE, there is a scientific need for radiobiological studies, which will allow informed treatment planning evaluations, and provide evidence to underpin an ethical plan for in vivo experiments. Ideally, experiments would be guided by a base of radiobiological studies, of which there are a limited number, due to the limitations discussed.

A collaboration between the University of Manchester (UK), and DESY (Germany) was initiated to attempt the in vitro irradiation of cancer cells using scanning methods. A radiobiology experiment measuring cell survival was the aim of the experiment, in an attempt to develop a VHEE irradiation protocol for further radiobiology experiments at DESY, as well as measuring the biological response to VHEE for the first time in cancer cells. This initial investigation into cancer cell survival was completed at the ARES RF linear accelerator with target energies of 100–155 MeV electrons, which was achieved following its finalised construction in 2021^14^. ARES demonstrated low energy jitter with a momentum stability of 6E-5 over a 16 h interval at 155 MeV^14^. The location of dedicated BSL-2 biology laboratory located in the nearby PETRA III experimental hall, as well as a highly stable VHEE beam meant that a protocol for the irradiation of cancer cells in vitro could be successfully developed.

## Results

### Dose uniformity

**Figure 1:**
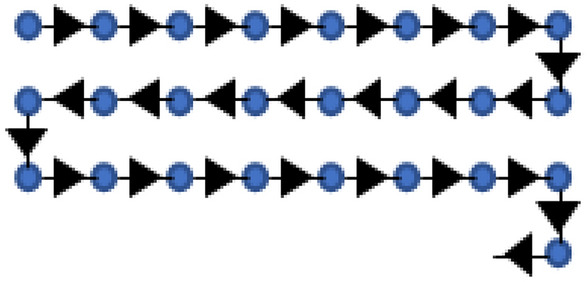
Example of the stage movement to irradiate using the ‘serpentine’ scanning pattern. The scanning pattern was used to create rectangular uniform dose fields over the sample area. Blue dots represent the irradiation spots and the black arrows represent direction of stage movement.

To obtain a uniform dose profile over the sample areas, various spot spacing’s (0.8–2.6 mm) were tested using a constant Gaussian beam with σ = 1.3 mm. It was decided that all samples should be irradiated in a ‘serpentine pattern’—an irradiation spot pattern that is represented in Fig. 1.

**Figure 2:**
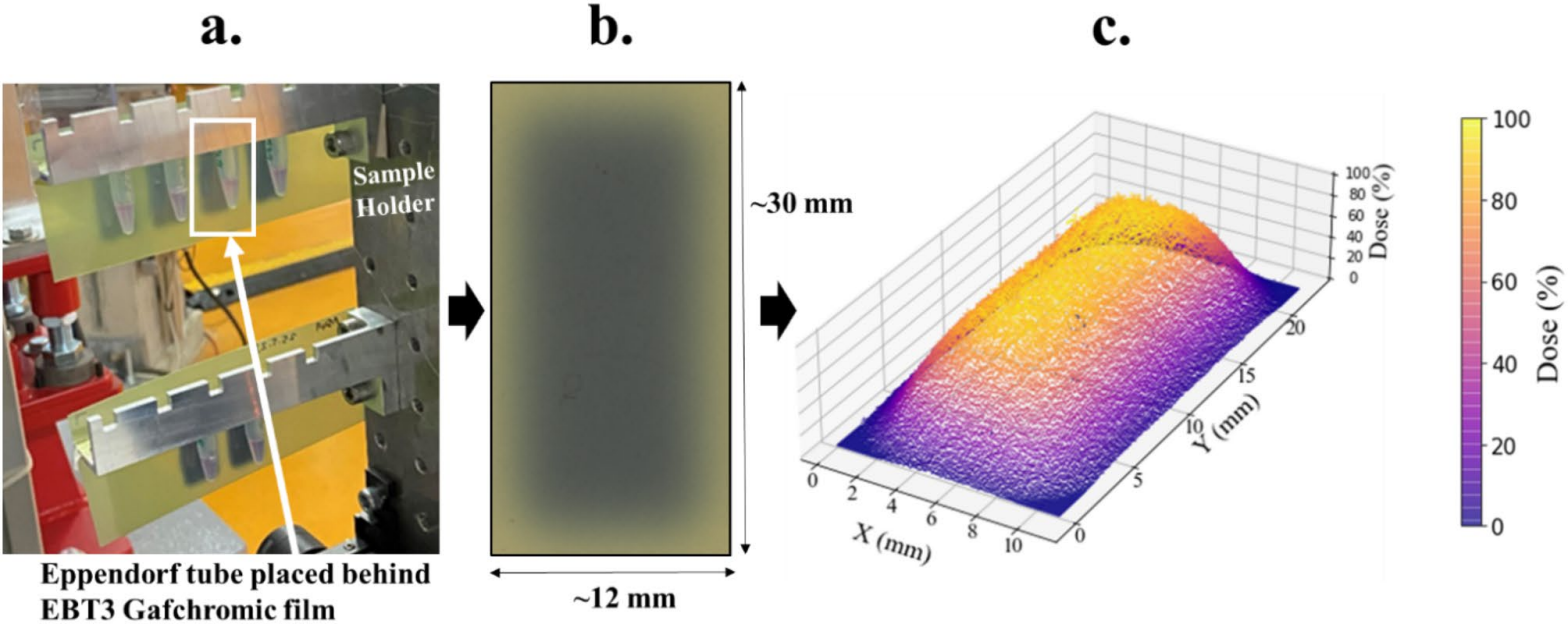
(**a**) Figure represents an example of the irradiated film pattern shown initially in the accelerator hall with irradiated samples. (**b**) Example of scanned film 24 h post irradiation. The scanned film is representative of the 7 × 12 irradiation pattern with 1.8 mm spot spacing that was used to irradiate all cell samples. The uniformity is indicated by the consistent darkening of the film within the rectangular area which is quantified further for all irradiated samples in Table 1. EBT3 Gafchromic film scanned using the Epson perfection V850 pro scanner. (**c**) A plot representing the percentage dose to sample. Average dose uniformity of the irradiated area is 4.54% (σ). A 3D representation of the scanned film image indicated. The pixel values from the scanned data have been converted to dose (%). X and Y axis indicates the size of the irradiated area.

1.8 mm spot spacing was quantified as having the highest dose uniformity based on X and Y dose profiles and standard deviation across all pixel values, based on EBT3 film data. This spot spacing was therefore used for irradiation of all samples. An example of this irradiation area using EBT3 Gafchromic film is indicated in Fig. 2.

Figure 2 provides a visual example of the uniformity, which has been quantified below in Table 1. The average dose for each irradiated sample area has been supplied for each individual sample. Average dose was measured as well as the standard deviation of pixels across the irradiated area of interest for each sample. Uniformity has been presented as the standard deviation from the mean (σ) across all pixels on the film for each sample irradiation (within the sample area of interest). These measurements show that the mean standard deviation across all samples is 4.54%, with a maximum deviation of 4.93 ± 1.25% for the lowest dose and a minimum of just 3.99 ± 0.60% for the 4.0 Gy dose point. It should also be noted that the dose uniformity of the EBT3 film itself is quoted between 2–3%, based on the manufacturers’ measurements^13^. Mean dose uniformity is also presented in respect to each dose where no trends are observed in correlation with increasing or decreasing average dose. Uniformity is therefore observed to be consistent between individual irradiations at each dose point to within 1.30% error. The homogeneity Index (defined by Eq. (1)) across samples ranged from 0.19 ± 0.02 (4.0 Gy) to 0.30 ± 0.08 (1.5 Gy). These results are consistent with the uniformity measurements, suggesting that the lowest dose is the least uniform, whereas the intermediate dose 4.0 Gy is the most.

**Table 1:** Average doses to each sample for each corresponding to the number of 18.3 pC pulses in each spot within the 7 × 12 irradiation pattern.

Number of 18.3 pC pulses	Dose (Gy)		Uniformity (%) (σ across all Pixels in Irradiated Area)		Homogeneity index	
	Mean	σ on Mean	Mean	σ on Mean	Mean	σ on Mean
1	1.5	0.1	4.93	1.25	0.30	0.08
2	2.5	0.2	4.19	0.83	0.28	0.06
3	3.2	0.3	4.72	1.30	0.27	0.08
4	4.0	0.1	3.99	0.60	0.19	0.02
6	6.0	0.3	4.56	0.98	0.25	0.05
7	6.7	0.4	4.87	0.91	0.24	0.02
Mean	N/A		4.54	0.98	0.26	0.05

The average dose represents the measured dose corresponding to the EBT3 film behind the sample, within the irradiated area. Note that the final calculated doses used in Fig. 4 and Tables 3 and 4 are slightly different due to the application of a factor that takes into account the dose change through the Eppendorf tube (more information in section “X-ray experimental setup on Xstrahl CIX3 cell irradiator at OCRB”). Dose uniformity indicates the standard deviation (σ) of dose across the irradiated area measured on the EBT3 film. Average doses and uniformity are measured using the red and green channels, with average dose representing the mean between the two channels, and uniformity representing the propagated standard deviation of the pixels in both red and green channels. Homogeneity Index was measured using an average of the red and green dose channels. Dose, uniformity and homogeneity index has been calculated for each dose, with the mean of the respective values and σ provided.

### Dose repeatability

**Figure 3:**
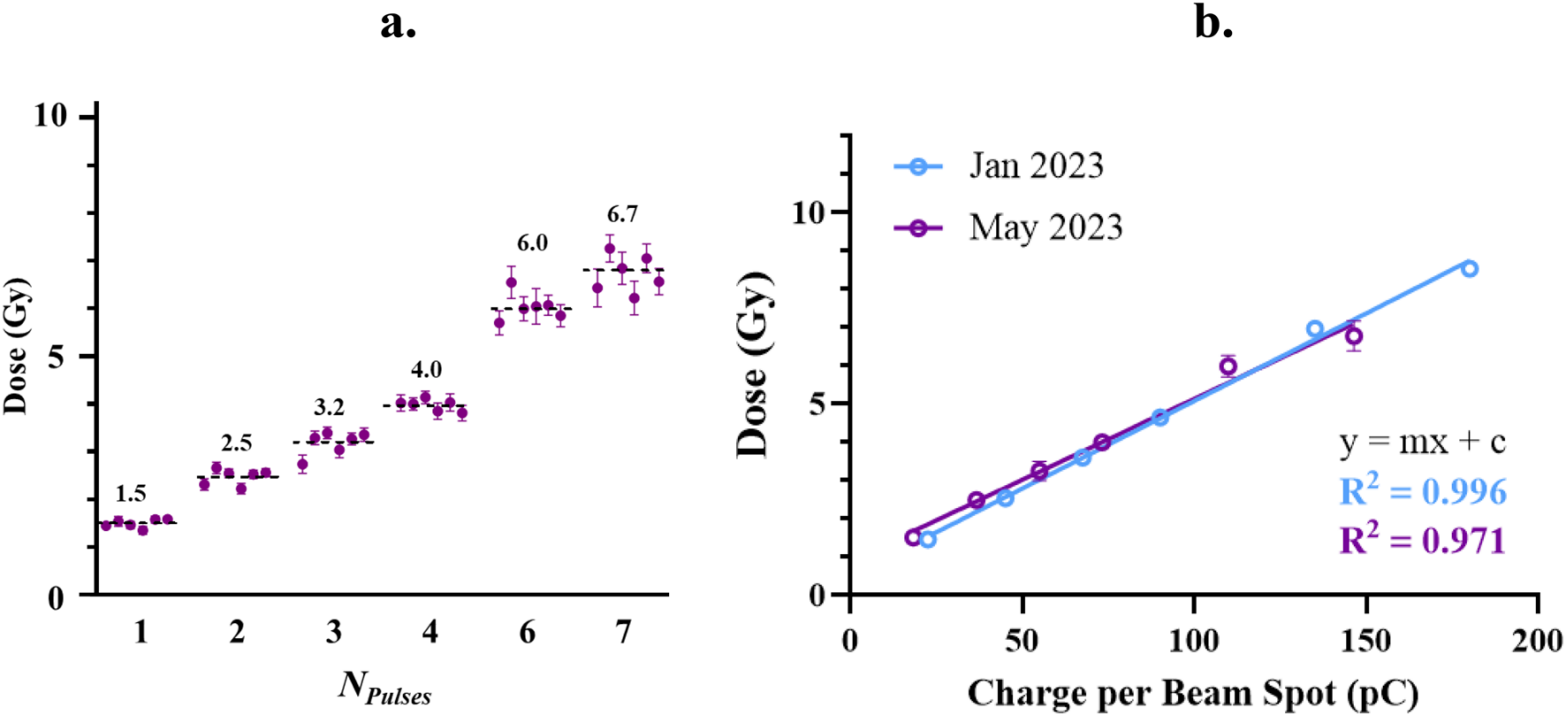
(**a**) All points represent measured dose from EBT3 Gafchromic film, within the irradiated sample area. Dose to sample was altered by the number of 18.3 pC electron pulses at each spot within the rectangular irradiation pattern. The number of pulses that corresponds to each dose is indicated on the x axis. Six repeats of each dose point was completed, represented by six separate points for each number of pulses. Error bars are indicative of the standard deviation across pixels in the measured area, specified in Table 1. The mean across six samples for each number of pulses is indicated by the dotted black line and corresponding black number. Points represent those that were measured in the May 2023 experimental run only. (**b**) Graph represents the dose measured from EBT3 Gafchromic film in response to increasing charge during experimental runs at ARES in both January and May 2023. Individual points represent mean values across six repeats and error bars are indicative of standard deviation measured across six irradiation repeats. During the January and May 2023 experimental runs, pulses with charges of 22.5 and 18.3 pC respectively were used, which is the factor responsible for the differing total charges between the two data sets.

Another critical factor was the ability to repeat specific doses to obtain experimental repeats that can be compared. This was tested by analysing mean dose to each individual sample for each dose and experimental repeat. Comparisons have also been made between two experimental runs several months apart (January and May 2023) where different beam charges were used. The dose repeatability was measured as mean dose ± standard deviation (σ) across six irradiated samples, for each supplied charge. The film measured doses were 1.5 ± 0.1, 2.5 ± 0.2, 3.2 ± 0.3, 4.0 ± 0.1, 6.0 ± 0.3 and 6.7 ± 0.4 Gy as shown in Fig. 3.

The correlation between charge and dose has been plotted with linear fits. Information regarding the fits is specified in Table 2 below.

**Table 2:** Linear fits to the data represented in Fig. 2b where the purple line represents measurements from January 2023, and the blue line represents measurements from May 2023.

Experimental run	*m*	*c*	R^2^
January 2023	0.046 ± 0.001	0.498 ± 0.145	0.996
May 2023	0.042 ± 0.001	0.897 ± 0.108	0.971
Significance between Linear Fits?	n/s p = 0.213	n/s p = 0.055	N/A
Pooled	0.043	0.832	N/A

Values for the fitting parameters for the y = mx + c linear equation and their associated error (σ) are indicated as well as a measure goodness of fit, R^2^. n/s = non-significant between linear fits to each data set. The pooled fitting parameters to the data across both January and May are also indicated due to the lack of significance between linear fits. It should be noted that due to the scanning pattern of the film, the charge vs dose fits are not expected to pass through zero. This is because each individual beam spot will have contributing dose from its neighbouring beam spot, even though this current is not considered on our X axis.

### Cell survival of A549 and PC3 cells in response to VHEE and X-ray irradiation

**Figure 4:**
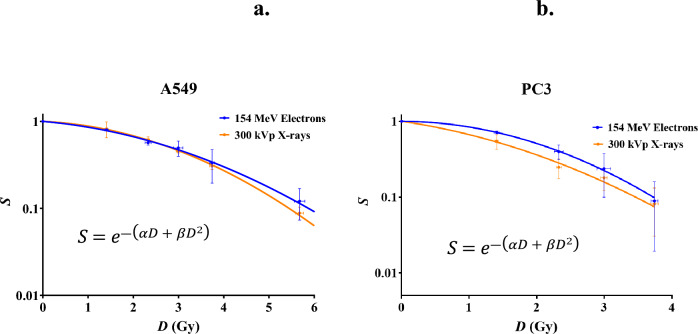
Curves indicate the proportion of cell survival (*S*) of A549 (**a**) and PC3 (**b**) cells in vitro in response to dose (*D*) of 154 MeV electrons and 300 kVp X-rays. Error bars are standard deviation where n = 3 for cell survival error and n = 6 for electron dose error. Fitted lines are the linear quadratic (LQ) model, with fitting parameters and goodness of fit indicated in Table 4.

A549 and PC3 cells were irradiated with doses of 154 MeV electrons, and 300 kVp X-rays, in matched experimental conditions. It was observed at higher doses that PC3 cells had low colony formation, so the cell survival in response to the two higher doses have not been indicated for this study. Results are presented below in Table 3 and Fig. 4.

**Table 3:** The proportion of surviving A549 and PC3 cells post-irradiation with X-rays and electrons of various doses.

Average dose ± σ (Gy)	Proportion of surviving cells			
	A549		PC3	
	300 kVp X-ray	154 MeV electron	300 kVp X-ray	154 MeV electron
1.4 ± 0.1	0.82 ± 0.17	0.80 ± 0.03	0.55 ± 0.12	0.71 ± 0.03
2.3 ± 0.2	0.61 ± 0.05	0.57 ± 0.04	0.25 ± 0.07	0.40 ± 0.09
3.0 ± 0.3	0.45 ± 0.02	0.50 ± 0.10	0.18 ± 0.06	0.24 ± 0.14
3.7 ± 0.1	0.31 ± 0.03	0.33 ± 0.14	0.08 ± 0.05	0.09 ± 0.07
5.7 ± 0.3	0.09 ± 0.01	0.12 ± 0.05	N/A	N/A
6.3 ± 0.4	0.07 ± 0.01	0.07 ± 0.03	N/A	N/A

Proportions are normalised to a surviving proportion of 1.0 for unirradiated samples. Average dose and standard deviation (σ) represents the dose measured across six repeats Eppendorf tube irradiations described further in Table 1. The two highest doses were not included in the final results for PC3 cells due to the high amounts of cell death resulting in difficulties accurately determining colony numbers.

Differences in cell survival were not found to be significant when using a two-way ANOVA test to compare between modalities at each specific dose for either cell line. The data is shown in Fig. 4, fitted to the linear quadratic (LQ) model.

### Measuring the relative biological effectiveness of VHEE

The RBE of VHEE was determined using values taken from the LQ fits to VHEE and X-ray cell survival data. Fitting parameters to the LQ models to each data set are detailed below in Table 4. Goodness of fit to the LQ model is also presented as well as D_0.5_ and D_0.1_, which represent the dose required to obtain 50% and 10% cell survival respectively. Values for VHEE RBE have been calculated from the D_0.5_ and D_0.1_ values to provide a quantification of the biological effectiveness of 154 MeV electrons in comparison to photons.

**Table 4:** Fitting parameters from LQ fits observed in Fig. 6 are indicated in this table for both cell lines and modalities as well as α/β ratios and measures for the goodness of fit.

	Fitting parameters and quantification of RBE			
	A549		PC3	
	300 kVp X-ray	154 MeV electron	300 kVp X-ray	154 MeV electron
α	0.06 ± 0.08	0.10 ± 0.07	0.30 ± 0.23	0.01 ± 0.18
β	0.07 ± 0.03	0.05 ± 0.03	0.11 ± 0.12	0.16 ± 0.09
α/β	0.84	2.13	2.82	0.06
R^2^	0.971	0.966	0.967	0.962
Adjusted R^2^	0.970	0.964	0.965	0.959
D_0.5_	2.82	2.85	1.51	2.03
VHEE RBE_0.5_	**0.99**		**0.74**	
D_0.1_	5.45	5.87	3.46	3.72
VHEE RBE_0.1_	**0.93**		**0.93**	
VHEE RBE_max_	1.67		0.03	
VHEE RBE_min_	0.85		1.21	

Significant values are in [bold].

Errors on the α and β fitting parameters represent 95% confidence intervals on the fits. D_05_ and D_0.1_ are measures of the dose required for endpoints of 50% and 10% cell survival respectively. These values were calculated from the relevant LQ model for each data set. The correlating VHEE RBE measurements were calculated the following way: (X-ray D_x_/VHEE D_x_). RBE_max_ refers to the α_VHEE_/α_X-ray._ RBE_min_ refers to the square root of β_VHEE_/β_X-ray_.

As indicated in Table 4, the RBE of VHEE can be observed to be 0.99 (D_0.5_) and 0.93 (D_0.1_) for A549 lung cancer cells, and 0.74 (D_0.5_) and 0.93 (D_0.1_) for PC3 prostate cancer cells. All sets of data were indicated to fit the LQ model with an R^2^ value > 0.95. α and β values varied significantly. A549 α values were 0.06 and 0.10 for X-rays and VHEE respectively. The respective X-ray and VHEE β values were 0.07 and 0.05, resulting in α/β ratios of 0.84 and 2.13. A major difference was in the α value for the PC3 cell line, with 0.30 (X-ray) and 0.01 (VHEE) calculated as the best fitting parameters available. Combined with β values of 0.11 and 0.16 for X-ray and VHEE respectively, this resulted in highly different α/β ratios of 2.83 and 0.06.

## Discussion

This study shows for the first time that cell cultures can be successfully irradiated with VHEE using a spot scanning method, to complete cell survival experiments. Dose uniformity across the irradiated sample area was measured to be 4.54% when using EBT3 Gafchromic films. This was considered to be a small error when also considering that the inherent uniformity error of the Gafchromic film is quoted to be 2–3%, in optimal conditions^15^. Gafchromic films have previously been studied to be a reliable dosimetry method for VHEE within their intended dose range^16,17^ and has been used for several experimental studies using VHEE thus far^5,10^. However, the film error is a limitation across all measurements in this study. Eventually, higher accuracy dosimetry could be achieved using ionisation chambers. Although not an issue for the dose rates used in this study, ultra-high dose rates do currently present a problem for standard chambers due to inefficient charge detection^18^. Developments such as the novel flashDiamond detector^19^ provide options to advance the precision and accuracy of dose to samples in an optimised experimental set up.

Separate from measuring accurate and precise dose to samples, repeatability of dose is one of the most important aspects of radiobiology. Dose to samples can vary significantly even with consistent beam parameters and conditions. Even small amounts of position jitter in the beam can change the obtained dose by a significant amount, especially when irradiating within a small area. Changes in amount and shape of dark current spots also needs to be considered and measured in experimental VHEE linac. High beam stability is required to obtain repeatable results and this was provided by the ARES linear accelerator, as well as low dark current throughout the experimental runs. To quantify repeatability, average dose to each sample was measured across six irradiated samples as well as the standard deviation from the mean (σ) of all pixel values in the irradiated area of interest as a measure of uniformity. Overall, the average standard deviation from the mean (σ) when combining all irradiation repeats at each dose is 4.54% This varies slightly between doses, with the 1.5 Gy having the largest standard deviation over six irradiated samples (4.93%) and the lowest standard deviation occurring at doses of 4.0 Gy (3.99%). Again, these values must be considered alongside the 2–3% dose error of the film.

This was determined as a successful response, however the dose error does limit the ability of radiobiologists to explore more nuanced responses to VHEE. For example, if we aim to explore and quantify differences in RBE that are most likely within a 0–10% difference of our reference modality, then a large number of studies will have to be performed to demonstrate statistical significance given typical dose uncertainty. The development of VHEE machines with highly stable beams for medical applications is an absolute requirement of clinical applications. Higher accuracy dosimetry for VHEE machines would be also be beneficial to improve on current radiobiological studies and drive clinical translation.

Another limitation of this study is fact that experiments across modalities were completed at different laboratories and times. RBE studies with VHEE would be more scientifically rigorous if there was availability to a photon reference modality in the same location. An ideal facility would allow scientists to perform comparable sets of experiments with X-rays alongside these with VHEE to have matched controls, timings, protocol and reduce inter-lab variation.

The spot scanning method was used to complete the irradiations, with the cells in suspension within 0.5 ml Eppendorf tubes. This method was chosen to maintain a small irradiation area (the serpentine pattern covered a ~ 10 × 20 mm area) and keep the irradiation time for each sample to under 5 min. This method could be utilised to cover larger areas such as flasks and well plates, however the considerably longer irradiation times would have to be taken into account, and the effect of this on the cells measured.

During the VHEE irradiation, cells remained in the accelerator hall for ~ 1 h. It must be considered that the Eppendorf tube environment is sealed and at room temperature, as well as the cells being in suspension. For these reasons, the same protocol was recreated for X-ray irradiated samples, with cells maintained in identical Eppendorf tubes for the same length of time. The effect of these environmental conditions were tested in unirradiated samples. Any effects on cell survival was measured using the plating efficiency for these unirradiated cells. There were no statistical differences between those cells plated immediately after counting, and those stored in suspension within the Eppendorf tubes. Plating efficiency had a larger variance in A549 cells than with PC3 cells, but no differences can be recognised between the two conditions. This test was critical for ensuring that using this alternate methodology was not introducing unpredicted levels of stress to the cells manifesting as the loss of proliferative capability, with could impact the overall result. The lack of difference between conditions was reassuring and the implication was that we could irradiate in the comparably small area of the 0.5 ml Eppendorf tube rather than a flask or well plate.

The cell survival was then measured in response to several doses and the LQ model was fitted to this data, as represented in Fig. 4. A high quality of fit to the LQ model indicated that both cell lines responded to both VHEE and X-ray irradiation as per the commonly described radiobiological model. The α/β values varied considerably between modalities, even in the case of A549 cells where the data points for VHEE and X-ray were noticeably similar. Due to the high goodness of fit of the LQ model to both cell lines and modalities, the fits were used to determine values for D_0.5_ and D_0.1_.

The quantification of VHEE RBE was completed by calculating D_0.5_ and D_0.1_, the dose required to kill 50% and 90% of cells respectively. The ratio of these doses was taken to calculate VHEE RBE values of 0.99 and 0.93 for A549 and 0.74 and 0.93 for PC3 cells. Average values for A549 and PC3 cells between the two conditions are 0.96 and 0.84 respectively, suggesting that the efficiency of VHEE cell killing is higher for lung cancer than prostate cancer in this case. Overall, the results indicate that VHEE have an RBE that is slightly less than, but close to 1.0. More investigations must be completed to add to the landscape of VHEE RBE.

Experimental investigations of VHEE RBE with plasmid DNA suggest an RBE of 1.1–1.2 (10). It is possible that the RBE > 1 for plasmids does not translate into a cancer cell model, and that the RBE for cell killing is closer to 0.9–1.0 based on the LQ fits. On the other hand, when measuring cell death at each dose point, there was no significant difference between VHEE and X-ray irradiated suggesting that RBE of VHEE is the same as that of photons. Our result is similar to another study investigating electron RBE using cell survival as the biological endpoint. Herskind et al.^20^ measured the RBE of 10 MeV electrons to be 0.98 and 0.91 for MCF7 (breast cancer) and HUVEC (endothelium) cells respectively, suggesting that electrons across a range of energies have an RBE of > 1 when measuring cell survival. An RBE value of 0.84 for cell survival has also predicted for electron energies in the 6–18 MeV range using Monte Carlo modelling^21^. It should be noted that clinically, an RBE of 1 is used for electrons and has been for several decades.

Micronuclei are markers of DNA damage and are commonly used to measure RBE. Micronuclei frequency has been used as a biological endpoint to predict electron RBE as 1.1–1.3 across three studies^22–24^ for electrons in the 1.5–8 MeV. Cell types measured were human lymphocytes and an ovarian cancer cell line. A recent systematic review of the literature did however highlight micronuclei frequency as an unreliable assay for quantifying biological effect between radiation modalities^25^. Naturally, more data is required as this is the first published response of cancer cells to VHEE and an overall picture of electron RBE is needed to predict biological effects accurately. Similar experiments with other cell types, including healthy cells, and eventually in vivo models is certainly required to fully understand the biological effect of VHEE.

## Methods

### Cell culture

A549 (human lung adenocarcinoma) and PC3 (human prostate adenocarcinoma) were cultured under sterile conditions in Roswell Park Memorial Institute (RPMI) 1640 medium (Gibco, 11875093) supplemented with l-Glutamine and 10% fetal bovine serum (FBS) (Gibco, 10270-106). Cells were cultured at 37 °C, 5% CO_2_.

Cell samples irradiated with 154 MeV electrons were cultured and prepared in the Biology Laboratory located in the PETRA III experimental hall, Deutsches Elektronen–Synchrotron (DESY) facility in Hamburg, Germany. In the case of cell samples irradiated with 300 kVp, cell culture and sample preparation took place in the Oglesby Cancer Research Centre (OCRB).

Cells were authenticated and routinely tested for mycoplasma contamination.

### Irradiation of A549 and PC3 cells in vitro

A549 and PC3 cells were irradiated with a range of doses across two research centres. Irradiations with 154 MeV electrons were completed at ARES and irradiations with 300 kVp X-rays were completed at the Oglesby Cancer Research Centre (OCRB), using an Xstrahl CIX3 cell irradiator.

Cells were prepared in suspension to a concentration of 5 × 10^5^ cells/ml in a 200 μl volume of cells. Cells were irradiated in 0.5 ml Eppendorf tubes (Eppendorf, 0030121023) at doses of 1.4, 2.3, 3.0, 3.7, 5.7 and 6.3 Gy for both 154 MeV electrons and 300 kVp X-rays. Three statistical repeats were completed for each dose and cell line. Physical beam parameters for the VHEE and X-ray irradiations are specified in Table 5 below.

**Table 5:** Physical parameters for the VHEE accelerator at ARES, and Xstrahl X-ray irradiator at the OCRB.

Modality	Electrons (VHEE)	X-ray
Energy	154 MeV	300 kVp
Beam/field size	Gaussian beam with σ ≃ 1.3 mm Scanned to a field size of ≃ 10 × 20 mm	Circular field, Diameter ≃ 30 cm
Average dose rate (calculated over scanned irradiation area for VHEE)	~ 1.8 Gy/s	2.13 Gy/min
Instantaneous dose rate (dose per pulse)	2 × 10^12^ (Gy/s)	N/A
Charge per pulse	18.3 pC	N/A
Repetition rate	10 Hz	N/A
Pulse length	800 fs	N/A
LET	0.61 keV/μm^26^	2–4.8 keV/μm for photons of similar energy (250 kVp)^27,28^

LET has been taken from stopping power tables for electrons in liquid water available at https://physics.nist.gov/cgi-bin/Star/e_table.pl. The X-ray LET range has been taken from a range of publications measuring LET of 250 kVp photons as this was the closest energy for which an LET estimate was observed.

Once samples were prepared, cells remained in suspension at room temperature for approximately 2 h including transport time to and from irradiation source, irradiation and seeding time. Figures referring to plating efficiency using this protocol, as well as images of colony formation are available in the Supplementary section of this manuscript.

### X-ray experimental setup on Xstrahl CIX3 cell irradiator at OCRB

0.5 ml Eppendorf tubes containing A549 or PC3 cells in suspension were irradiated with 300 kVp X-rays by lying tubes flat on the internal turntable within the Xstrahl CIX3 cell irradiator. The turntable ensured uniform irradiation over the samples from the vertical X-ray source. Dose to samples was measured based on the X-ray exposure time, with a dose rate of 2.13 Gy/min ± 0.8% used. A 0.7 mm copper filter was used.

### Electron experimental set up at ARES

**Figure 5:**
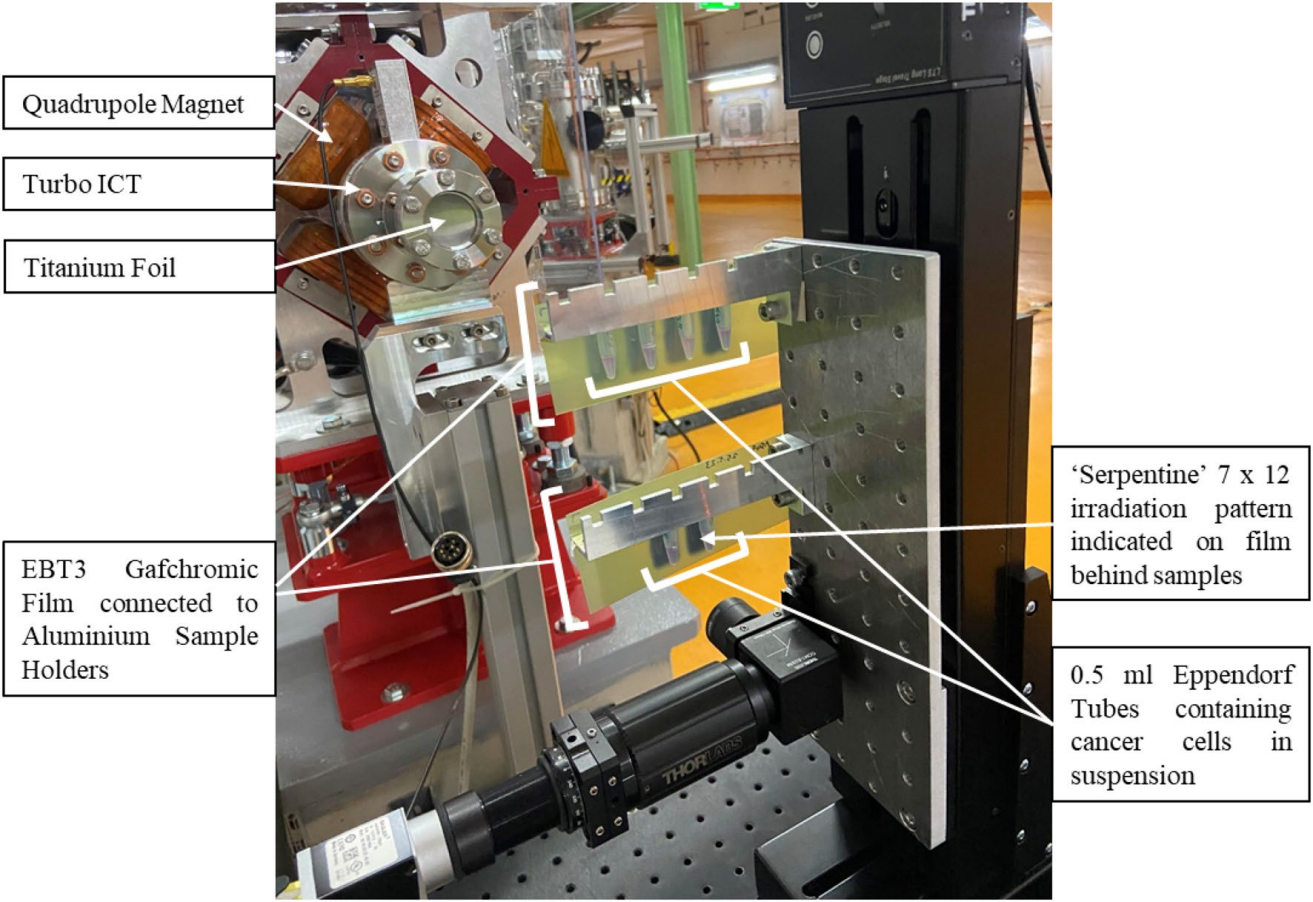
A labelled photograph of the experimental area during the May 2023 run after irradiating cancer cells. Significant components of the experimental area are indicated including the sample location, the aluminium sample holder and EBT3 Gafchromic film for measurement of dose to samples. Note that during the irradiation, an identical rectangular section of EBT3 Gafchromic film was placed behind the sample, but has been removed here for visibility of the Eppendorf tubes and sample holder.

**Figure 6:**
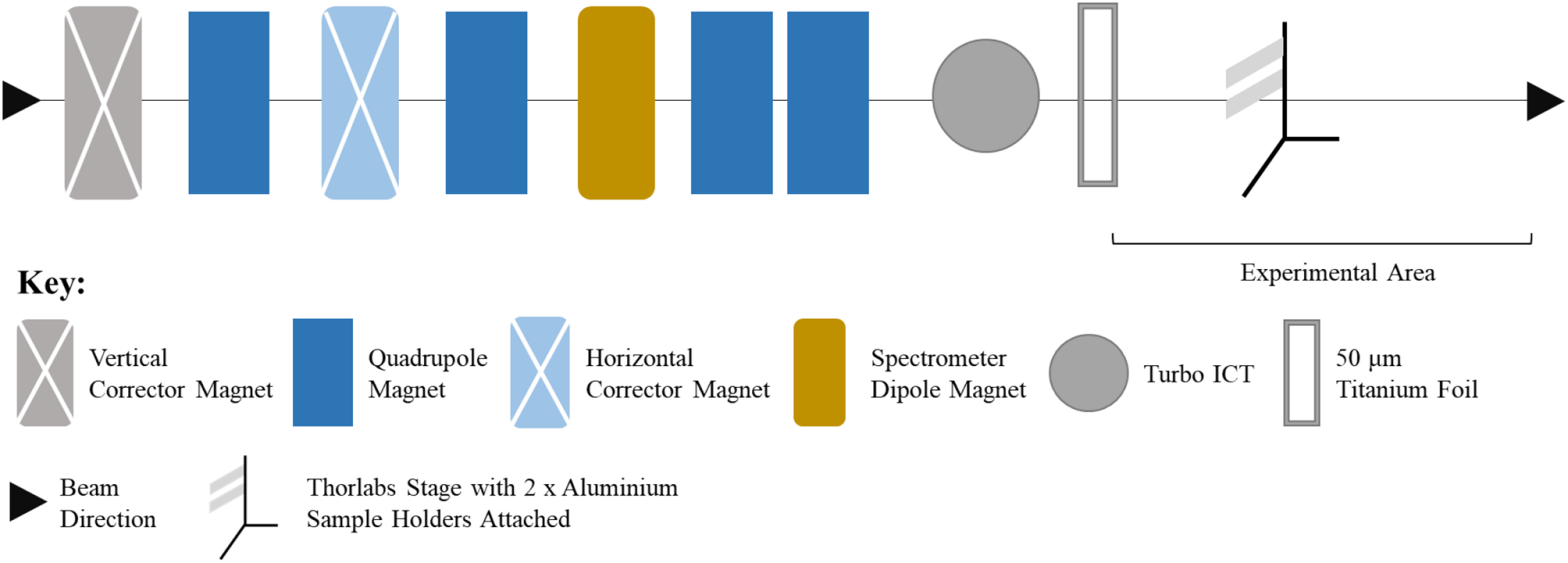
A schematic representation of the ARES beamline is indicated. Electrons are generated by a normal conducting RF photoinjector, and are then accelerated using an S-band system. Focussing and steering of the beam are provided by several quadrupole magnets, as well as a dipole and corrector magnets. Current measurements are provided by the turbo integration current transformer (ICT). A 50 μm thick Titanium foil separates the accelerator vacuum from air. The electrons then terminate in the experimental area at an energy of approximately 154 MeV.

Cells were prepared and irradiated in 0.5 ml Eppendorf tubes and transported from the Biology Laboratory to the ARES accelerator hall in a polystyrene box. Samples were loaded in the custom made C250 aluminium sample holder as indicated in Fig. 5. Rectangles of EBT3 Gafchromic film were secured in front and behind the samples in the irradiated area to measure dose for each irradiation. The sample holder was attached to a Thorlabs translation stage (Thorlabs, LTS300/M) to ensure precise movements of the samples, therefore creating a uniform scanned dose over the Eppendorf tube volume. Figure 6 shows the schematic of the beamline as well as the samples in the experimental area.

Samples were irradiated in a pre-optimised scanning pattern that consisted of overlapping Gaussian beam spots, achieved by the movement of the stage in a ‘serpentine’ pattern. Beam size was maintained at 1.3 mm σ for all experiments and the scanning pattern consisted of a 7 × 12 spot pattern using 1.8 mm spot spacing. Beam charge was maintained at 18.3 pC per pulse. Dose to samples was altered by varying the number of pulses administered at each spot in the 7 × 12 pattern. 1, 2, 3, 4, 6, and 7 pulses per spot corresponded to doses of 1.4, 2.3, 3.0, 3.7, 5.7 and 6.3 Gy respectively. Post-irradiation, the accelerator hall was accessed immediately, cells were removed and transported to the biology laboratory for processing.

### Measuring cell survival using clonogenic method

Cell survival was measured in both cell lines using a clonogenic assay. Cells were seeded into six well plates within 1 h post-irradiation. Three seeding densities were used per dose, with each seeding density prepared in duplicate, using pre-optimised seeding densities. Cells were then incubated for 8 (A549) or 11 (PC3) days at 37 °C, 5% CO_2_. After the incubation time, cells were washed with PBS and colonies were fixed and stained with 0.7% crystal violet solution (Sigma–Aldrich, V5265) prepared in 30% methanol (Fisher Scientific, M/4000/21). Colonies were counted, with a colony defined a cluster of > 50 cells.

### Dosimetry

The Xstrahl machine for irradiations with 300 kVp X-rays was calibrated twice per annum to current national standards by the Christie Medical Physics team using an ionisation chamber. Ionisation chamber and probes are calibrated by Christie Medical Physics team annually. At the time of writing, the most recent dosimetry checks measured the X-ray dose rate at 2.13 Gy/min, with a percentage error of 0.8%. Collating dosimetry data from the previous 2 years shows that the maximum percentage error on the dose is 1.3% which has therefore been used to plot the X-ray error bars in Fig. 4. Dose measurements were also completed using EBT3 Gafchromic film to validate average dose and uniformity of the irradiation field.

Dosimetry of VHEE at ARES was completed by simulating the dose delivered for a given charge using TOPAS Monte Carlo simulation (version 3.7.0)^29,30^, with validation using EBT3 Gafchromic film. EBT3 film was calibrated using a medical 15 MeV electron linac at the Christie Hospital, Manchester, UK. All calibration and reference films were scanned on an Epson perfection V850 pro scanner (Epson, B11B224401) at 300 dpi. Measured dose refers to the average of red and green colour channels in every instance.

Film was placed directly in front of and behind samples to measure dose received in the irradiated region directly behind the Eppendorf tube. The difference between the measured dose behind and in front of the tube was calculated to be 6.3%, which was applied uniformly to the dose measured behind the sample to calculate values for the dose received by the sample volume.

Dose uniformity in the irradiated area was measured as the standard deviation across all pixels on the EBT3 film within the irradiated area, as measured using Image J software.

The homogeneity index was calculated using the equation:

1 $$HI = \left( {P_{max} - P_{min} } \right)/\left( {P_{max} + P_{min} } \right)$$

where HI is the homogeneity index, *P*_*max*_ and *P*_*min*_ are the maximum and minimum pixel dose on the Gafchromic film in the sample area.

Dose repeatability was calculated by measuring the standard deviation (σ) of the average doses of six individually irradiated samples, with access to the accelerator hall in between each irradiation.

### Fitting of radiobiological model to cell survival data

The linear quadratic (LQ) model was fitted to cell survival data in response to radiation dose. The equation,

2 $$S = e^{{ - \left( {\alpha D + \beta D^{2} } \right)}}$$

where* S* is the proportion of surviving cells, D represents Dose (Gy) and α (Gy^−1^) and β (Gy^−2^) are both fitting parameters that are described further for each data set in Table 4. All fits were completed using GraphPad Prism (version 8) software.

### Statistical analysis

A Student’s paired* t* test was used to compare between the unirradiated plating efficiencies for two plating methods.

A two way analysis of variance (ANOVA) was applied to the irradiated cell survival for A549 and PC3 datasets separately to determine differences between radiation modalities at each dose. This was followed up by a Sidak’s multiple comparisons test to identify statistical differences between VHEE and X-ray cell survival. *p*-values < 0.05 were considered to be statistically significant.

The statistical analysis for both tests was completed using GraphPad Prism (version 8). The threshold for statistical significance used throughout was *p* < 0.05.

### Supplementary Information

Supplementary Information.

## Data Availability

The data underlying this article is available in the article, presented in table format throughout. Any other data or specific information underlying this article will be shared on reasonable request to the corresponding author.
