# Supplementary material for: First in vitro measurement of VHEE relative biological effectiveness (RBE) in lung and prostate cancer cells using the ARES linac at DESY
Source: Sci Rep. 2024 May 13;14:10957. doi: 10.1038/s41598-024-60585-7 (PMC11091057; doi:10.1038/s41598-024-60585-7)
Supplement: Supplementary file 1 — Supplementary Information. [file 41598_2024_60585_MOESM1_ESM.docx]

**11. Supplementary**

**Cell survival of A549 and PC3 cells in response to experimental conditions**

During the incubation period, the cells were observed under the microscope to successfully form colonies at each dose point. An example of this is shown in Fig. 4 for unirradiated cells and cells irradiated with 2.3 Gy.


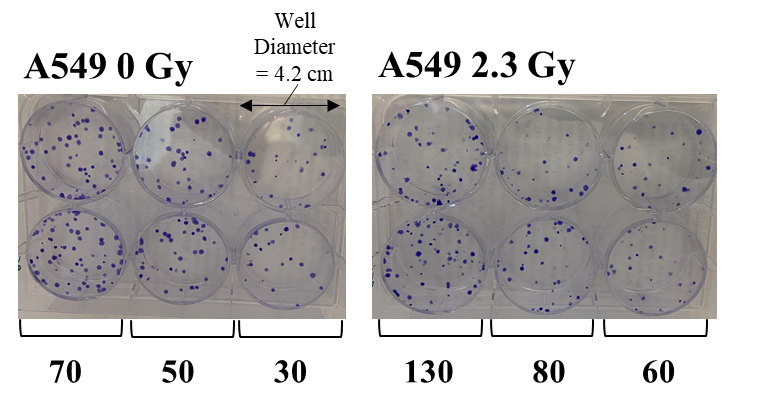


Supplementary Figure 1. Successful colony formation observed in A549 cells 9 days after seeding during the experimental run at ARES, DESY. Images represent examples of colony formation post-irradiation with 2.3 Gy (right) of VHEE, as well as in an unirradiated control (left). Numbers represent the number of cells seeded per well of the 6 well plate. As can be observed from the image, each seeding density was seeded in duplicate. Total size of each 6 well plate is approximately 13.0 x 8.5 cm. Colonies were stained with crystal violet. A cluster of more than 50 cells was considered a colony.

During the course of the VHEE irradiations, samples were in suspension, at room temperature in a sealed Eppendorf tube. This experimental design was therefore tested against a protocol that involved immediate plating of cells after counting, to ensure that these unfavourable conditions did not have an impact on plating efficiency, and therefore cell survival. This is represented in Supplementary Fig. 2, with cell survival (%) indicated for both cell lines in experimental conditions, and with plating instantly post-counting.

1. **b.**

**
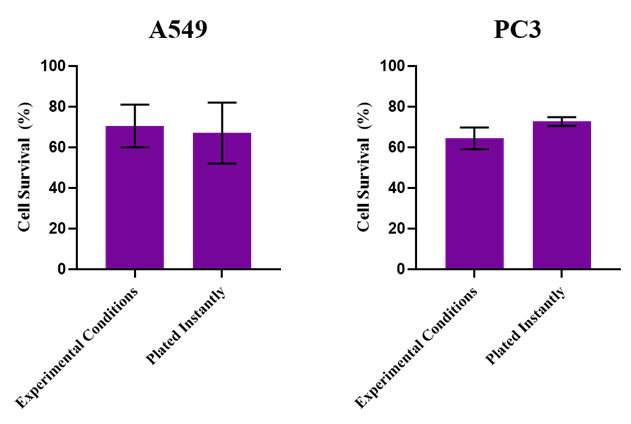
**

Supplementary Figure 2. Plating efficiency (measured as percentage of surviving cells) of A549 (a) and PC3 cells (b) is indicated in unirradiated cells in response to two protocols. All results were taken from samples plated and incubated at the Oglesby Cancer Research Centre (Manchester). ‘Experimental conditions’ refers to the experimental method used during the cell survival experiment, as described in the methods section. This involves the cells being contained within an Eppendorf tube, in suspension and at room temperature for ~2 hrs. ‘Plated instantly’ condition refers to cells plated immediately in the six well plate post-counting, without any intermediate transfer to Eppendorf tubes. Error bars are the standard deviation (σ) across three experimental repeats in every case.

Average (± σ) plating efficiency of A549 cells was represented by 70.5 ± 18.1% and 67.0 ± 25.9% cell survival for experimental and immediate plating conditions respectively. PC3 cells demonstrated 64.3 ± 9.3% and 72.6 ± 3.8% cell survival for experimental and immediate plating conditions respectively. A Student’s paired t-test indicated that there were no significant differences between conditions for either cell line (A549, *p* = 0.889) and (PC3, *p* = 0.364).
